# Supplementary figures and images for: How stimulation frequency and intensity impact on the long-lasting effects of coordinated reset stimulation
Source: PLoS Comput Biol. 2018 May 10;14(5):e1006113. doi: 10.1371/journal.pcbi.1006113 (PMC5963814; doi:10.1371/journal.pcbi.1006113)

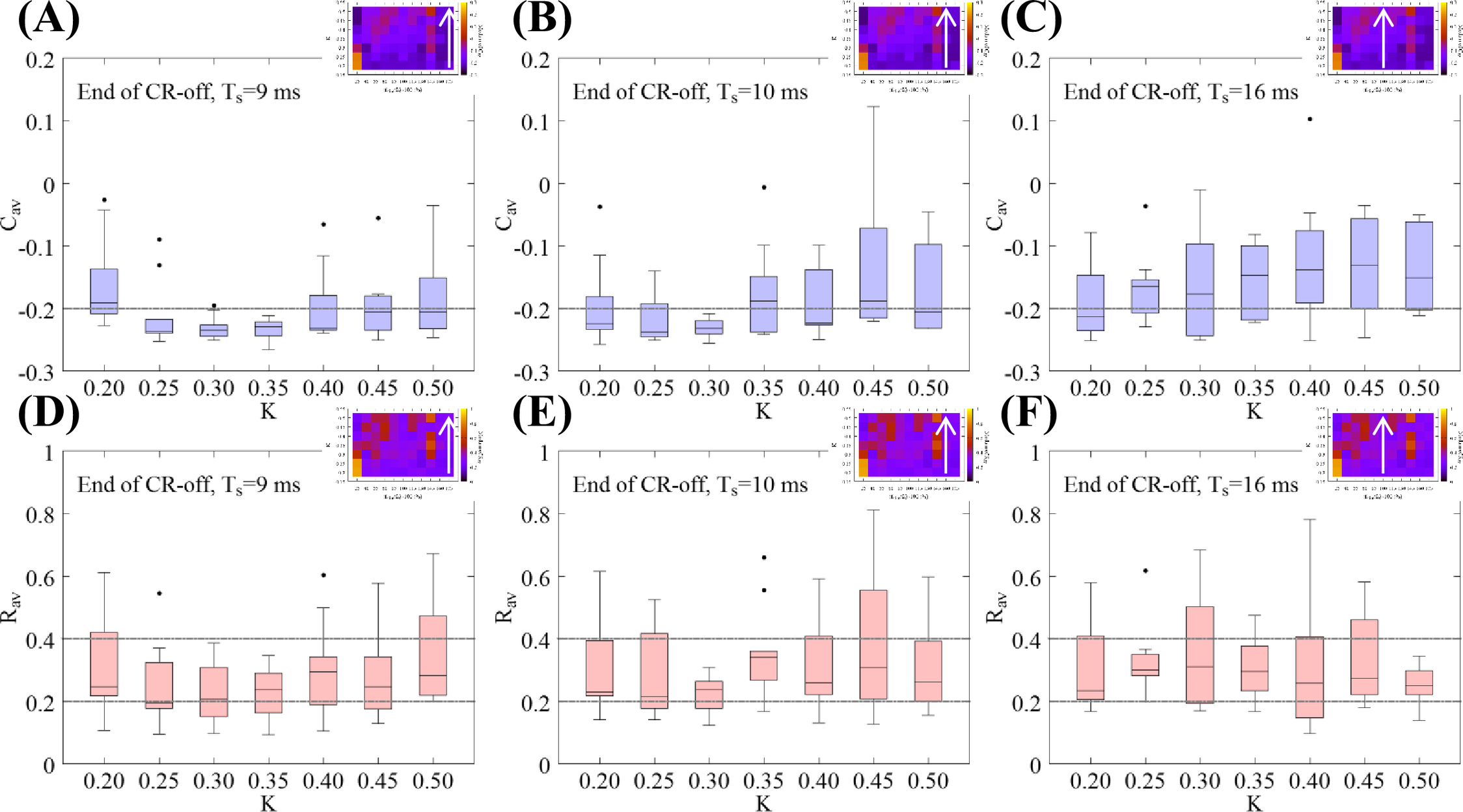

Supplement: S1 Fig — (A) Cav and (D) Rav for Ts = 9 ms. (B) Cav and (E) Rav for Ts = 10 ms. (C) Cav and (F) Rav for Ts = 16 ms. These three period values are indicated by the white arrows in the inset Cav and Rav general median overview color-plots. The dotted horizontal line(s) (one for the Cav and two for the Rav boxplots) are visual cues to facilitate comparison between different panels. (TIF) [file pcbi.1006113.s001.tif]

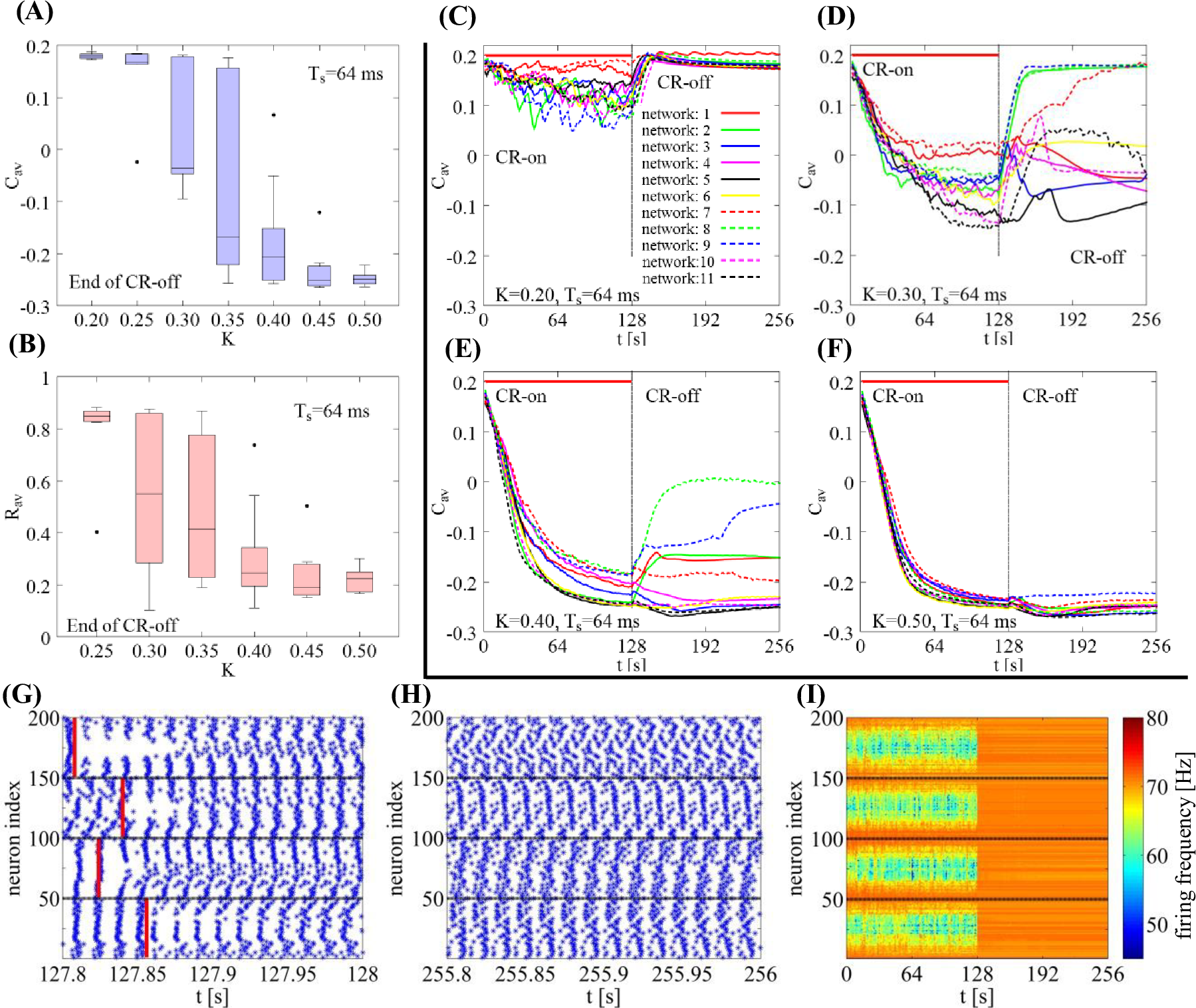

Supplement: S2 Fig — (A) Boxplots of Cav for the different K values and (B) boxplots for Rav. (C)- (F) Time evolution of Cav for K = 0.20, 0.30, 0.40, 0.50 and 11 different initial networks and signals. (G) Raster plot at the end of the CR-on period for K = 0.50 and network 1. (H) Raster plot at the end of the CR-off period. Blue points indicate the spiking times (horizontal axis) of each neuron (vertical axis), the red vertical lines point out the onset of each CR stimulation at the center of these sub-intervals (neurons i = 25, 75, 125, 175). (I) Firing frequencies of the individual neurons for K = 0.50 (network 1). The horizontal black lines (G, H and I) are visual clues to distinguish between the four stimulated neuronal subpopulations. (TIF) [file pcbi.1006113.s002.tif]

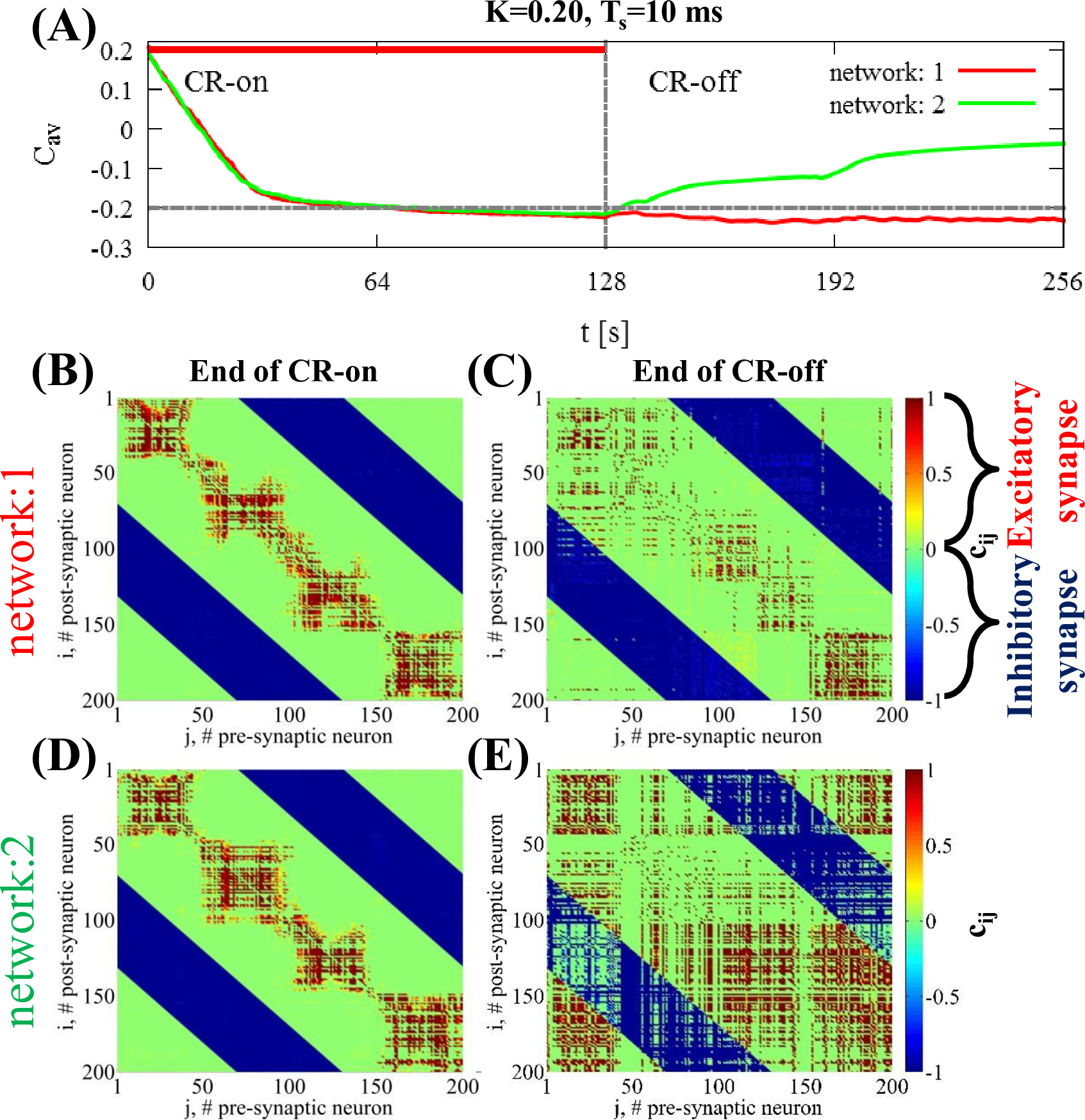

Supplement: S3 Fig — (A) The time evolution of the mean synaptic weight Cav and the corresponding connectivity matrices at the end of the CR-on period [panels (B) and(D)] and CR-off period [panels (C) and (E)] for two different network initializations and random RVS CR sequences: for network 1 [red solid line in (A) and connectivity matrices (B), (C)] and network 2 [green solid line in (A) and connectivity matrices (D), (E)] for K = 0.20, Ts = 10 ms. (TIF) [file pcbi.1006113.s003.tif]

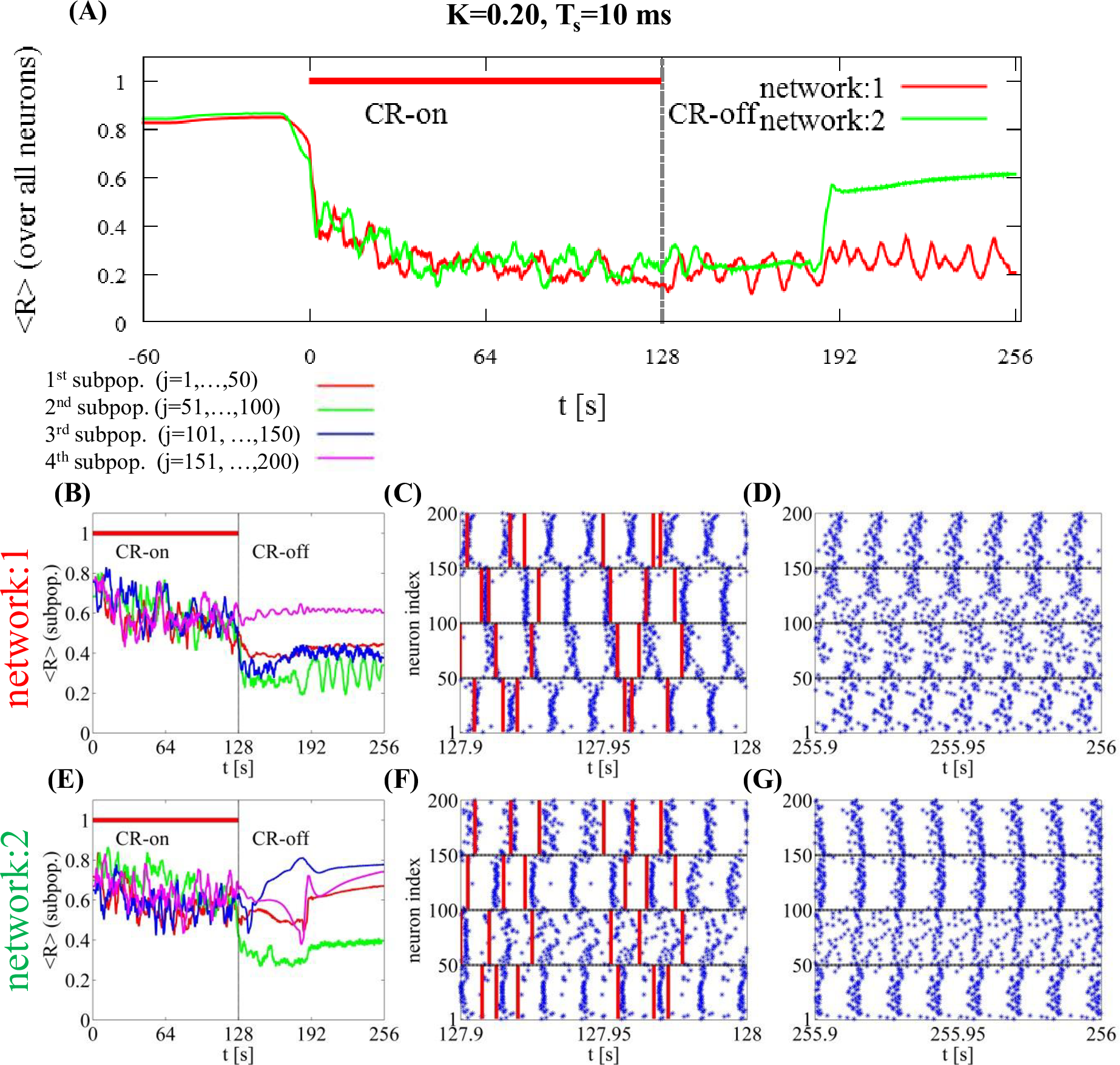

Supplement: S4 Fig — (A) Moving average of the order parameter < R > (averaged over a sliding window of 20 ms width and K = 0.20, Ts = 10 ms) for the whole network and for the four subpopulations of networks 1 (B) [belonging to network 1 of (A)] and 2 (E) [network 2 of (A)]. The definition of the four subpopulations reflects the equidistant delivery of CR stimulation and the corresponding order parameters are coded by different color (see inset). In the raster plots at the end of the CR-on period (C), (F) and at the end of the CR-off period (D), (G), blue points indicate spiking times (horizontal axis) of each neuron (vertical axis), red lines illustrate the onset of each CR stimulus at the center of that neuronal subpopulation. Horizontal black lines delineate the allocation of the four neuronal sub populations. (TIF) [file pcbi.1006113.s004.tif]

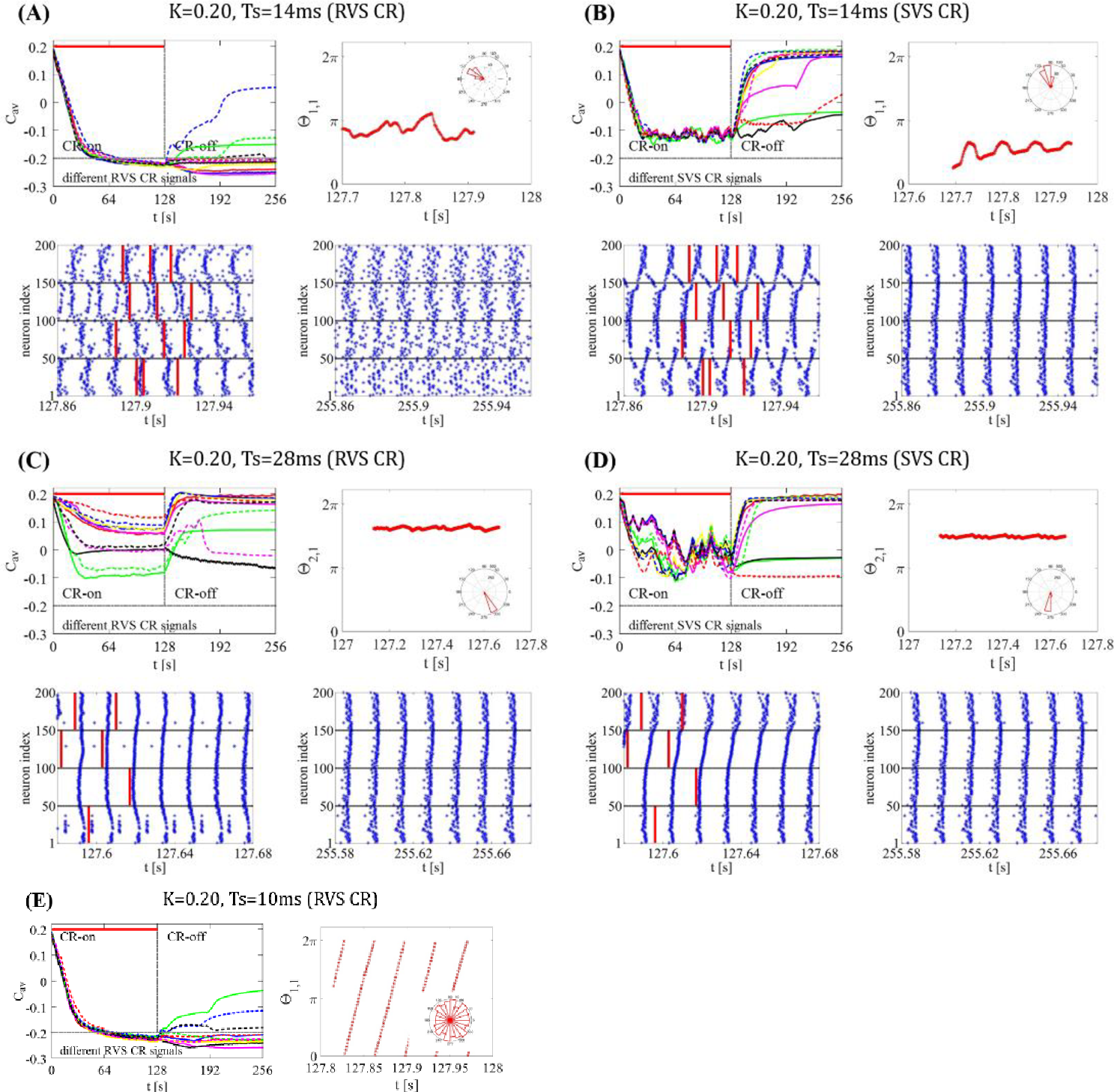

Supplement: S5 Fig — Investigation of phase locking effects for Ts = 14 ms (1:1 resonance) Ts = 28 ms (1:2 resonance) for CR intensity K = 0.20. Each panel is composed of four subpanels showing the time evolution of the mean synaptic weights Cav (top left sub-panels for each (K,Ts)-parameter pairs and all 11 networks). The top right sub-panels show the time evolution of the Θn,m(t) while in the inset figure its distribution is plotted. The two bottom sub-panels show the raster plots (format as in S4 Fig) at the end of the CR-on period (bottom-left panels) and at the end of the CR-off period (bottom-right panels). Panel (E) presents a typical example for an optimal parameter pair (K,Ts) = (0.20,10) for RVS CR in this case. (TIF) [file pcbi.1006113.s005.tif]
