# Supplementary material for: How stimulation frequency and intensity impact on the long-lasting effects of coordinated reset stimulation
Source: PLoS Comput Biol. 2018 May 10;14(5):e1006113. doi: 10.1371/journal.pcbi.1006113 (PMC5963814; doi:10.1371/journal.pcbi.1006113)
Supplement: S1 Text — (DOCX) [file pcbi.1006113.s006.docx]

**Relationship between Average Synaptic Weight and Average Synchrony**

In S1 Fig we focus on the three prominent vertical stripes connected with favorable stimulation outcome (indicated by the white arrows in the insets of S1 Fig), where the median values for both mean synaptic weight and order parameter are small, indicating weak excitatory connectivity and synchrony. Along all these three stripes CR induces an anti-kindling (see S1A-C Figs) and a long-lasting desynchronization (see S1D-F Figs). For $T_{s}=9 ms,10$ms the impact of CR is stronger compared to $T_{s}=16$ ms and leads to weaker synaptic connectivity and further reduction of synchrony. However, the latter difference does not translate to the amount of synchrony. In fact, for some $K$ values the period $T_{s}=16$ ms we observe a more desynchronized state than $T_{s}=9 ms,10$ ms (S1D-F Figs). Put otherwise, pronounced differences of the average synaptic weight need not be reflected by pronounced differences of the average amount of synchrony.
