# Supplementary material for: How stimulation frequency and intensity impact on the long-lasting effects of coordinated reset stimulation
Source: PLoS Comput Biol. 2018 May 10;14(5):e1006113. doi: 10.1371/journal.pcbi.1006113 (PMC5963814; doi:10.1371/journal.pcbi.1006113)
Supplement: S2 Text — (DOCX) [file pcbi.1006113.s007.docx]

**Intensity Dependent Stimulation Effects**

At the stimulation frequency $f_{\mathrm{stim}}=145\%f_{0}$ ($T_{s}=11$ ms) for larger stimulation intensities no pronounced desynchronization is achieved, and acute effects (Fig 3) as well as sustained after-effects are poor (Figs 4A and 4B). A qualitatively different phenomenon occurs at the lowest stimulation frequency $f_{\mathrm{stim}}=25\%f_{0}(T_{s}=64$ms$)$: The anti-kindling effects get more pronounced with increasing stimulation intensity. S2 Fig presents a detailed analysis for different stimulation intensities$K$, where S2A and S2B Figs depict boxplots of $C_{av}$ and $R_{av}$ for the different $K$ values. A clear inverse *sigmoidal*-like decay is observed in both figures indicating a monotonic-like gradual tendency to more effective long-lasting anti-kindling as the CR intensity increases for this particular stimulation period. The time evolution of $C_{av}$ for each different network is shown in S2C-F Figs for$K=0.20, 030, 0.40, 0.50$. The most striking result is presented in S2F Fig with $K=0.50$ where we observe an optimal impact of CR stimulation, i.e. all networks/signals reach a rather low value and maintain this low mean synaptic weight value until the end of the CR-off period.

S2G and S2H Figs show the raster plots at different time windows (each of 200 ms width) for the $K=0.50$ (network 1) case of S2F Fig, at the end of the CR-on and at the end of the CR-off period respectively. The horizontal black lines are visual cues, distinguishing the four separately stimulated groups of neurons. CR stimulation is administrated at neurons $i=$25, 75,125,175. S2I Fig depicts the impact of the CR stimulation, again for $K=0.50$ (network 1), on the firing frequencies (color bar) of the network, calculated in time windows of length$100\cdot T_{s}$. Let us recall (see *Simulation description* section) that the intrinsic fire rate before the CR is ~71 Hz. Hence, the CR stimulation, in this particular effective case, causes a decrease in firing rate to ~60 Hz (greenish range).
