# Supplementary material for: How stimulation frequency and intensity impact on the long-lasting effects of coordinated reset stimulation
Source: PLoS Comput Biol. 2018 May 10;14(5):e1006113. doi: 10.1371/journal.pcbi.1006113 (PMC5963814; doi:10.1371/journal.pcbi.1006113)
Supplement: S3 Text — (DOCX) [file pcbi.1006113.s008.docx]

**Predictability of Long-Term Outcome and Mechanism of CR Stimulation on the Presence of STDP**

This section serves to illustrate two findings: (i) Macroscopically similar states at the end of the CR-on period may lead to qualitatively different long-term dynamics. (ii) Neither prominent features of the synaptic connectivity matrix at the end of the CR-on period nor the dynamics of the selectively stimulated subpopulations at the end of the CR-on period enabled us to predict the long-term outcome.

To investigate the impact of different initial network conditions and different realizations of RVS CR sequences on the long-lasting sustained effects, we consider one parameter pair ($K,T_{s}$) and two different network initializations. For both networks 1 and 2 the mean synaptic weight$C_{av}$ reaches similar, low values by the end of the CR-on period (S3A Fig) with similar connectivity matrices (S3B and S3D Figs). Nevertheless, in both cases the long-term outcome is qualitatively different concerning both the mean synaptic weight $C_{av}$ (S3A Fig) and the corresponding connectivity matrices (S3C and S3E Figs).
