# Supplementary material for: How stimulation frequency and intensity impact on the long-lasting effects of coordinated reset stimulation
Source: PLoS Comput Biol. 2018 May 10;14(5):e1006113. doi: 10.1371/journal.pcbi.1006113 (PMC5963814; doi:10.1371/journal.pcbi.1006113)
Supplement: S4 Text — (DOCX) [file pcbi.1006113.s009.docx]

**Predictability of Long-Term Outcome and Mechanism of CR Stimulation on the Presence of STDP**

In addition, we consider the amount of synchrony of the entire neural network and its subpopulations as well as the corresponding raster plots for the same set of parameters as in S3 Fig. S4A Fig shows the moving average of the order parameter $<R>$ (in a running window of $400\cdot T_{s}$ length) for the whole neuron ensemble. We use moving averages because of the presence of strong fluctuations of $R$. At $t=0$s both networks are strongly synchronized ($R\approx0.85$) and rapidly undergo a CR-induced desynchronization. After the cessation of CR stimulation, both networks maintain a similar degree of global desynchronization for up to $t \sim$190 s, when only one of them starts getting more synchronized (green solid line, network 2). CR is delivered at four stimulation sites. To specifically analyze the impact of CR on the subpopulations in the vicinity of the stimulation sites, we calculated the time average of the order parameter for each subpopulation, too.

In the second and third row of S4 Fig we show the time averaged order parameter $<R>$ for each subpopulation. The allocation to subpopulations is given by the stimulation setup, as CR stimuli are equidistantly delivered to four different sites. The subgroups’ order parameters are indicated by different colors (S4B and S4E Figs) for network 1 (red solid line in S4A Fig) and 2 (green solid line in S4A Fig) respectively. The amount of synchrony within the four subpopulations is similar during stimulation, but quite different post-stim. In network 1 three of the subpopulations remain in a desynchronized state post-stim (S4B Fig) and the corresponding neurons do not fire coincidently (S4D Fig). In contrast, three of the subpopulations of network 2 resynchronize (S4E Fig), as reflected by the coincident spiking of their neurons (S4G Fig).

This example is intended to illustrate the multitude of dynamic states evolving from comparably similar states at stim offsets. These different states differ with respect to extent of synchrony within subpopulations, their mutual phase relationship and time courses of these quantities. We found several parameter pairs ($K,T_{s}$) with similar acute, but qualitatively sustained long-lasting effects. For these different parameter pairs, we performed similar comparisons as in the example above. Macroscopic quantities like $C_{av}$ and $<R>$ of the entire network or of the subpopulations together with the subpopulations’ mutual phase relationships did not enable us to find markers predictive of specific long-term outcome in cases with a plurality of CR-off responses originating from similar end of CR-on states.

Intriguingly, RVS CR stimulation does not induce phase resets of the individual subpopulations that are time-locked to the corresponding stimuli (S4C and S4F Figs). Rather, the time differences between the stimulus onsets (red bars in S4C and S4F Figs) and the neurons’ spikes (blue dots in Figs S4C and S4F) do not cluster around a preferred value. This finding was repeatedly observed in a portion of parameter pairs $\left( K,r_{0} \right)$.
