# Supplementary material for: How stimulation frequency and intensity impact on the long-lasting effects of coordinated reset stimulation
Source: PLoS Comput Biol. 2018 May 10;14(5):e1006113. doi: 10.1371/journal.pcbi.1006113 (PMC5963814; doi:10.1371/journal.pcbi.1006113)
Supplement: S5 Text — (DOCX) [file pcbi.1006113.s010.docx]

**Spiking Dynamics, Resonances and Phase Entrainment Analysis**

In this we section, we perform a more detailed analysis on the spiking dynamics for two characteristic cases where resonance effects may take place. Namely, for $T_{s}$=14 ms and $T_{s}$=28 ms (RVS & SVS CR), we perform a phase entrainment analysis of the phase $\psi\left( t \right)$ of the order parameter defined by the equation:

$$R\left( t \right)e^{i\psi\left( t \right)}=N^{-1}\sum_{j} e^{i\varphi_{j}\left( t \right)}$$

and the CR phase $\phi_{\mathrm{CR}}\left( t \right)$. The latter increases linearly in time from 0 to $2\pi$ with a stimulation cycle of length$T_{s}$. We then plot the $n:m$ phase difference modulo $2\pi$ defined by

$\Theta_{n,m}\left( t \right)=\left[ n\psi\left( t \right)-m\phi_{\mathrm{CR}}\left( t \right) \right]_{mod(2\pi)}$.

In S5 Fig, we plot the time evolution of the mean synaptic weights $C_{av}$ (top left sub-panels for each ($K,T_{s}$)-parameter pairs and all 11 networks). The top right sub-panels show the time evolution of the $\Theta_{n,m}\left( t \right)$ (for *network #1*) while in the inset figure its distribution is plotted. The two bottom sub-panels show the raster plots at the end of the CR-on period (bottom-left panel) and at the end of the CR-off period (bottom-right panel). The red lines illustrate the onset of each CR stimulus at positions$i=25, 75,125,175$. The presence of strong entrainment due to the phase resonance $1:2$ of mean ensemble phase and the CR phase is confirmed for $T_{s}$=28 ms for both RVS & SVS CR (S5C,D Fig). For $T_{s}$=14 ms, there is also an entrainment effect which is more pronounced for SVS CR (S5B Fig). In the RVS CR case, it seems that the stimulus has succeeded to decrease the mean synaptic weights $C_{av}$ more efficiently during the CR-on period which results in a long-lasting desynchronization for the majority of the networks/initializations (S5A Fig). In S5E Fig, we show one typical example for an optimal parameter pair ($K,T_{s}$) $=(0.20,10)$ for RVS CR in this case.
